# Localization of the neuropeptides pituitary adenylate cyclase-activating polypeptide, vasoactive intestinal peptide, and their receptors in the basal brain blood vessels and trigeminal ganglion of the mouse CNS; an immunohistochemical study

**DOI:** 10.3389/fnana.2022.991403

**Published:** 2022-10-26

**Authors:** Anne Marie Lund, Jens Hannibal

**Affiliations:** ^1^Faculty of Health and Medical Sciences, Institute of Clinical Medicine, University of Copenhagen, Copenhagen, Denmark; ^2^Department of Clinical Biochemistry, Faculty of Health Sciences, Bispebjerg and Frederiksberg Hospital, University of Copenhagen, Copenhagen, Denmark

**Keywords:** PACAP, VIP, PAC1, VPAC1, VPAC2, CGRP, migraine, trigeminal ganglion

## Abstract

Pituitary adenylate cyclase-activating polypeptide (PACAP) and vasoactive intestinal peptide (VIP) are structurally related neuropeptides that are widely expressed in vertebrate tissues. The two neuropeptides are pleiotropic and have been associated with migraine pathology. Three PACAP and VIP receptors have been described: PAC1, VPAC1, and VPAC2. The localization of these receptors in relation to VIP and PACAP in migraine-relevant structures has not previously been shown in mice. In the present study, we used fluorescence immunohistochemistry, well-characterized antibodies, confocal microscopy, and three-dimensional reconstruction to visualize the distribution of PACAP, VIP, and their receptors in the basal blood vessels (circle of Willis), trigeminal ganglion, and brain stem spinal trigeminal nucleus (SP5) of the mouse CNS. We demonstrated a dense network of circularly oriented VIP fibers on the basal blood vessels. PACAP nerve fibers were fewer in numbers compared to VIP fibers and ran along the long axis of the blood vessels, colocalized with calcitonin gene-related peptide (CGRP). The nerve fibers expressing CGRP are believed to be sensorial, with neuronal somas localized in the trigeminal ganglion and PACAP was found in a subpopulation of these CGRP-neurons. Immunostaining of the receptors revealed that only the VPAC1 receptor was present in the basal blood vessels, localized on the surface cell membrane of vascular smooth muscle cells and innervated by VIP fibers. No staining was seen for the PAC1, VPAC1, or VPAC2 receptor in the trigeminal ganglion. However, distinct PAC1 immunoreactivity was found in neurons innervated by PACAP nerve terminals located in the spinal trigeminal nucleus. These findings indicate that the effect of VIP is mediated via the VPAC1 receptor in the basal arteries. The role of PACAP in cerebral arteries is less clear. The localization of PACAP in a subpopulation of CGRP-expressing neurons in the trigeminal ganglion points toward a primary sensory function although a dendritic release cannot be excluded which could stimulate the VPAC1 receptor or the PAC1 and VPAC2 receptors on immune cells in the meninges, initiating neurogenic inflammation relevant for migraine pathology.

## Introduction

Pituitary adenylate cyclase-activating polypeptide (PACAP) and vasoactive intestinal peptide (VIP) belong to the secretin/glucagon neuropeptide superfamily (Vaudry et al., 2009). The discovery of PACAP was based on its ability to stimulate cyclic AMP production in rat anterior pituitary cells and led to the first isolation from ovine hypothalamic extracts (Miyata et al., 1989). Two bioactive forms exist: a 27 and 38 amino acid form produced from the same precursor protein. Of the two, PACAP38 is the more prevalent form. PACAP shows high homology (∼68%) (Hirabayashi et al., 2018) with VIP, a 28-amino acid peptide first isolated from the porcine intestine and capable of inducing profound vasodilation (Harmar et al., 2012). Both PACAP and VIP are well-conserved neuropeptides with identical amino acid structures across vertebrate species (White et al., 2010; Harmar et al., 2012). PACAP and VIP are pleiotropic neuropeptides abundant in both the central and peripheral nervous systems. PACAP immunoreactivity has been found in many tissues including the gastrointestinal tract (Hannibal et al., 1997b), respiratory tract (Uddman et al., 1991), reproductive organs (Fahrenkrug and Hannibal, 1996), and pancreas (Fridolf et al., 1992; Hannibal and Fahrenkrug, 1999). It is believed to play a cytoprotective and anti-inflammatory role (Elekes et al., 2011; Toth et al., 2020). Double immunostaining has shown that PACAP often colocalizes with the sensory capsaicin-sensitive calcitonin gene-related peptide (CGRP) in nerve fibers (Fahrenkrug and Hannibal, 1996; Hannibal et al., 1997b). VIP has been discovered in many of the same tissues as PACAP, often in different nerve fibers, including the cerebral blood vessels of several species (Edvinsson et al., 1980; Fahrenkrug et al., 2000).

Neurovascular control involves nerve fibers from sympathetic nerves, parasympathetic postganglionic nerve fibers from the sphenopalatine and otic ganglion, and sensory nerve fibers originating in the trigeminal ganglion (Edvinsson, 2002). VIP nerve cell bodies innervating cerebral blood vessels are known to originate from parasympathetic ganglia in rats (Suzuki et al., 1988; Hara et al., 1989). PACAP nerve cells have been demonstrated in both sympathetic ganglia, parasympathetic ganglia (Sundler et al., 2006), and in the sensory trigeminal ganglion where it colocalizes with CGRP (Eftekhari et al., 2015; Frederiksen et al., 2018). PACAP binds G-protein-coupled receptors PAC1, VPAC1, and VPAC2 (Moldovan Loomis et al., 2019). VPAC1 and VPAC2 signal through cAMP-PKA pathways, whereas the PAC1 receptor also stimulates the PKC pathway (Harmar et al., 2012). PACAP and VIP bind VPAC1 and VPAC2 with equal affinity, while PAC1 has a thousandfold higher affinity for PACAP and is therefore considered a PACAP-specific receptor (Harmar et al., 2012; Hannibal et al., 2017b; Edvinsson et al., 2018). mRNA of all three receptors has been found in human cerebral arteries and the trigeminal, otic, and superior cervical ganglia, but the detailed location has not been shown (Knutsson and Edvinsson, 2002).

Several sensory neuropeptides involved in neurovascular control and inflammation have been associated with migraine pathology including CGRP, PACAP, and VIP. All three neuropeptides have a strong vasodilative effect and the ability to trigger delayed migraine-like attacks in nearly all migraine patients (Schytz et al., 2009; Hansen et al., 2010; Amin et al., 2014; Guo et al., 2017; Pellesi et al., 2021). Experimental studies in humans have shown that PACAP and CGRP can induce vasodilation after a 20-min infusion (Lassen et al., 2008; Schytz et al., 2009). The vasodilation peaked after the end the infusion and lasted for several hours. Delayed migraine-like attacks occurred 4–5 h after a 20-min infusion of PACAP or CGRP (Schytz et al., 2009; Hansen et al., 2010; Amin et al., 2014). Two hours infusion of VIP was necessary to trigger migraine attacks and the period of vasodilation was shorter (Pellesi et al., 2021). A CGRP monoclonal antibody (eptinezumab) has recently been approved for prophylactic treatment of frequent episodic and chronic migraine in adults (Datta et al., 2021), while a PACAP monoclonal antibody is in early-phase clinical development (Brevig et al., 2021). The PAC1 receptor has been the primary receptor in focus due to its high affinity to PACAP and studies indicating a role of this receptor in pain sensation (Jongsma et al., 2001; Davis-Taber et al., 2008). However, monoclonal antibodies for the PAC1 receptor offer no benefit over placebo in migraine prevention (Ashina et al., 2021).

More insight into the neuroanatomy of PACAP, VIP, and their receptors is needed. No previous studies have shown this in detail in mice. Mice have become a new tool in the understanding of anatomy, physiology, and behavior, due to genetic modification (knock-out or knock-in). It has developed our understanding of new mechanisms as well as new therapeutic strategies. This study will examine the distribution of PACAP, VIP, and their receptors in the basal blood vessels (circle of Willis), trigeminal ganglion, and brain stem spinal trigeminal nucleus (SP5) of the mouse CNS using well-characterized antibodies for PACAP (Hannibal et al., 1995), VIP (Fahrenkrug et al., 1995), CGRP, PAC1 (Hannibal et al., 2017b), VPAC1 (Fahrenkrug et al., 2000), and VPAC2 (Hannibal et al., 2017b).

## Materials and methods

### Animals

**FIGURE 1:**
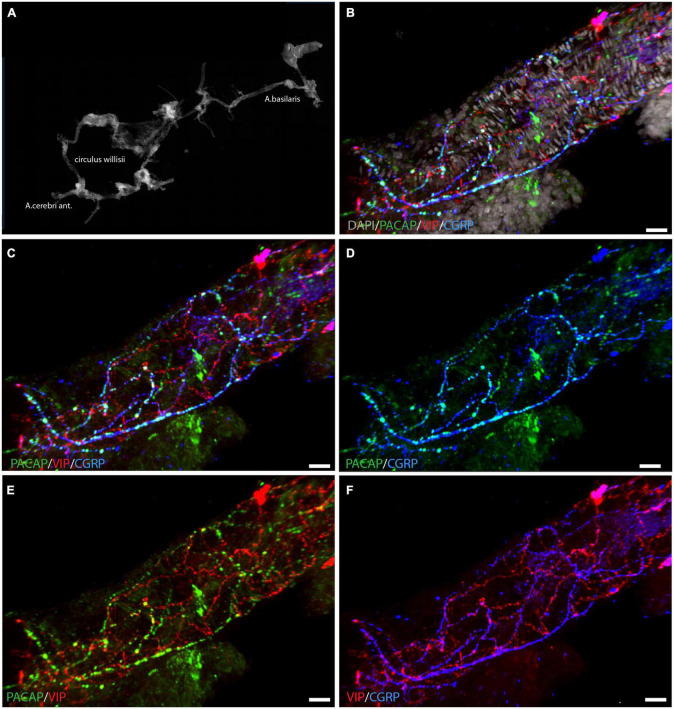
Localization of PACAP (green), VIP (red), and CGRP (blue) in whole mount preparation of a cerebral artery. An overview of the circle of Willis is seen in panel **(A)** with DAPI-staining. A segment of a cerebral artery is seen in panels **(B–F)** with immunostaining of PACAP, VIP, and CGRP in different combinations. Triple immunostaining is shown in panels **(B,C)**, note that PACAP and CGRP are seen in the same nerve fibers, illustrated in panel **(D)**. Panels **(E,F)** visualize the different distribution patterns of PACAP/VIP **(E)** and CGRP/VIP **(F)**. Panels **(B–F)** represent a 3D reconstruction of a Z-stack (Z = 0.5 μm) of 117 digital sections. Scale bars: **(B–F)** = 20 μm.

Twenty-six mice, 13 males and 13 females, were killed by decapitation [Zeitgeber time (ZT) 3–6, ZT = 0, lights ON] and used for immunostaining. Two mice of each genotype, lacking either the VPAC2 or the PAC1 receptor, were used to verify the specificity of staining (Hannibal et al., 2017a; Supplementary Figure 1). All mice were kept in a 12-h light dark (LD) cycle with free access to food and water at the Department of Clinical Biochemistry at Bispebjerg Hospital. Animals were treated according to the principles of Laboratory Animal Care (Law on Animal Experiments in Denmark, LBK NR474, May 15, 2014) and Dyreforsoegstilsynet, Ministry of Justice, Denmark, who issued the license number 2017/15-020101364 to Jens Hannibal, thereby approving the study. The brains were rapidly extracted after decapitation. The basal arteries (Figure 1A) and trigeminal ganglia were removed by careful dissection under a stereomicroscope followed by fixation overnight in Stefanini fixative (2% PFA, picric acid). The trigeminal ganglia were then placed in 30% sucrose overnight, frozen on dry ice and cut in a cryostat in 12 μm thick sections prior to freezing (−80°C). The basal arteries were stored in cryoprotectant at −20°C until immunohistochemically processed. The brain stems from two perfusion-fixed mice, dehydrated in 30% sucrose and frozen before (Hannibal et al., 1997a) were cut into 40 μm sections and stored in cryoprotectant until further processing.

### Immunohistochemistry

Immunohistochemical staining was performed on whole mounts of basal arteries and brain stem sections, and sections from the trigeminal ganglia of the mouse. All antibodies used in the study are listed in Tables 1, 2. The tissues were treated in heat-induced antigen retrieval at 80°C with EnVision Flex GV805 in distilled water pH 6 for 1 h and 30 min. The tissues were then washed (PBS/0.25% Triton X-100), blocked for endogenous peroxidase activity (1% H_2_O_2_ in 1× PBS), and subsequently blocked with 5% normal donkey serum (Jackson Immunoresearch Laboratories) to avoid non-specific staining. Hereafter, the tissues were incubated with primary rabbit/sheep polyclonal antibodies (Table 1). Two primary antibodies raised in the same species were applied using a combination of biotinylated tyramide (Akoya Biosciences) and streptavidin-conjugated Alexa Fluor dyes (Jackson ImmunoResearch) followed by incubation of the second primary rabbit antibody which was detected using Envision (DAKO) and tyramide-conjugated Alexa Fluor dyes (Invitrogen, Thermo Fisher Scientific) as previously described (Hannibal et al., 2017b). PACAP-38 (523C-5, RRID:AB_2650426) (Table 1) was amplified using biotinylated tyramide (Table 2), while PAC1 (35J8, RRID:AB_2814675), VPAC1 (4G28, RRID:AB_2814929), and VPAC2 (623S, RRID: 2814676) receptor antibodies (Table 1) were amplified with Envision (Hannibal et al., 2017b) (Table 2). VIP (291E, RRID:AB_2313759) (Table 1) was visualized with Alexa Fluor 594-conjugated donkey IgG (Table 2). For triple immunostaining, a third primary antibody from a different species (sheep) was mixed in the same buffer as the primary rabbit antibody and visualized with the Cy5-conjugated donkey IgG (Table 2). Sections and cerebral arteries were mounted in Glycerol/PBS 1:1/DAPI 1:1000. Immunohistochemical control procedures are described in Supplementary material and Supplementary Figures 1–4.

**TABLE 1:** Primary antibodies.

Antibody	Code #	Host	Source	Dilution	RRID
PACAP-38	523C-5	Rabbit	KBA-BBH	1:100,000	AB_2650426
VIP	291E-3	Rabbit	KBA-BBH	1:1,000	AB_2313759
PAC1	35J8-9	Rabbit	KBA-BBH	1:10,000	AB_2814675
VPAC1	4G28	Rabbit	KBA-BBH	1:2,000	AB_2814929
VPAC1	4G28	Rabbit	KBA-BBH	1:10,000	AB_2814929
VPAC2	623S	Rabbit	KBA-BBH	1,20,000	AB_2814676
CGRP	PC205L	Rabbit	Sigma-Aldrich	1:500	AB_2068524
CGRP	22560	Sheep	Abcam	1:2,000	AB_725809

**TABLE 2:** Secondary antibodies.

Antibody	Code #	Species	Source	Dilution
Biotinylated tyramide	SAT700001EA		AKOYA Biosciences	1:100
EnVision+	K4002	Rabbit	DAKO	1:2
Alexa Fluor 488 donkey IgG	A11015	Rabbit	Invitrogen, Thermo Fisher	1:200
Alexa Fluor 594 donkey IgG	A21207	Rabbit	Invitrogen, Thermo Fisher	1:1,500
Alexa Flour 488 tyramide	B40953		Invitrogen, Thermo Fisher	1:250
Alexa Flour 594 tyramide	B40957		Invitrogen, Thermo Fisher	1:250
Cy5 streptavidin	016-170-084		Jackson ImmunoResearch	1:500
Alexa Fluor 488 streptavidin	016-540-084		Jackson ImmunoResearch	1:500
Cy5 streptavidin	016-170-084		Jackson ImmunoResearch	1:500
Biotin-SP donkey IgG	711-065-152	Rabbit	Jackson ImmunoResearch	1:1500
Cy5 donkey IgG	713-175-147	Sheep	Jackson ImmunoResearch	1:250

### Photomicrography, colocalization, and cell counting

The analysis was performed using an iMIC confocal microscope equipped with appropriate filter settings for detecting DAPI, Cy2/Alexa488, Cy3/Alexa594, and Cy5/Alexa647. The following objectives were used: X10, numerical aperture (NA) = 0.35; X20, NA = 0.75; X40, NA = 1.3; and X60, NA = 1.46. The highest resolution *r* = λ/*NA*, where λ is the imaging wavelength, was for X60 = 174 nm. The iMIC used an Andromeda spinning disk system for confocal imaging and a 16-bit camera (model C10600-10B-H, Hamamatsu Photonic) for recording. The iMIC microscope had a 12-bit camera (model C84484-03G02, Hamamatsu Photonic) for wide-field microscopy. Images in Z-stacks were deconvoluted in AutoQuant X3 (Media Cybernetics) before being analyzed in IMARIS 9.9.0 (Oxford Instruments). For 3D analysis, the basal blood vessels and brain sections were photographed using the X20, X40, or X60 objectives. Images were obtained as tiles and Z-stacks, and these were stitched together using the LA Stitch plug-in in Fiji software version 1.53q (NIH, USA).

The cell counting module in Fiji was used to determine whether PACAP and CGRP were colocalized in the same cells. Cells expressing CGRP were marked, followed by the marking of PACAP-positive cells. Hereafter, cells colocalizing both peptides were determined in the mouse trigeminal ganglion. Only immunoreactive cells with visible nuclei were counted. Four different mice were chosen for cell counting with one or two ganglia per animal. Images were taken at 20× magnification and stitched together in Fiji. The results are summarized in Table 3. All images were adjusted for contrast and brightness in Fiji or IMARIS and mounted into plates in Adobe Illustrator CS5 (Adobe).

**TABLE 3:** Cell counting.

Measure	M-1*	M-2	M-3	M-4	Mean	Std.	SEM
Total immunoreactive cells, #	147	128	179	132	146.5	23.2	11.6
Only CGRP, #	94	96	98	91	94.8	3.0	1.5
Only PACAP, #	2	1	5	1	2.3	1.9	0.9
Colocalized, #	51	31	76	40	49.5	19.5	9.7
Only CGRP, % of total	63.9	75.0	54.7	68.9	65.7	8.6	4.3
Only PACAP, % of total	1.4	0.8	2.8	0.8	1.4	1.0	0.5
Colocalized, % of total	34.7	24.2	42.5	30.3	32.9	7.7	3.8

*M-1 to M-4 refers to mouse 1–4.

## Results

### Vasoactive intestinal peptide, pituitary adenylate cyclase-activating polypeptide, and receptors in basal arteries

**FIGURE 2:**
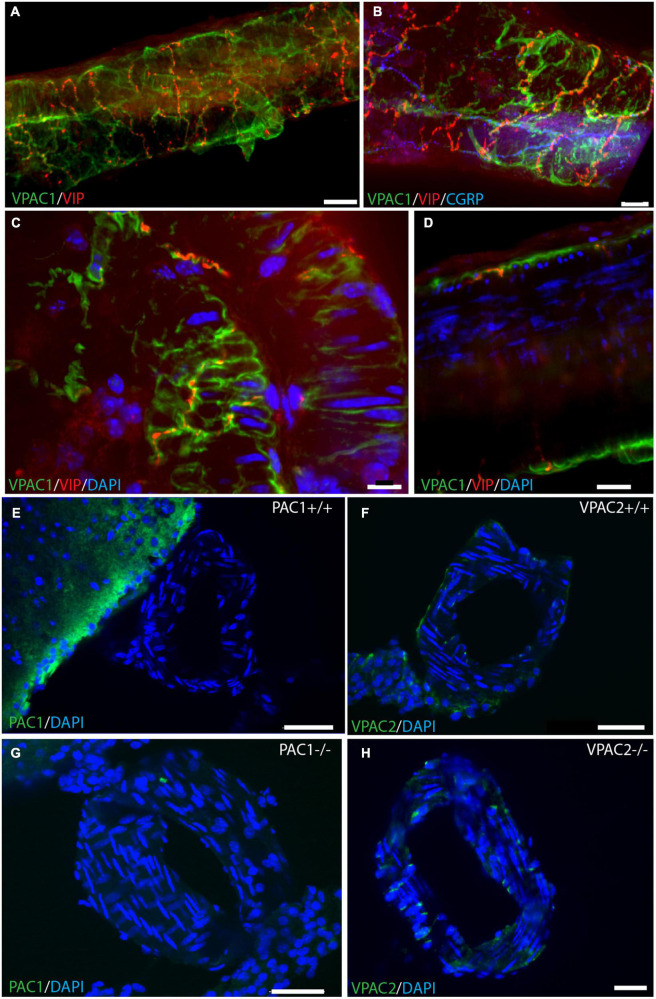
VPAC1 receptor immunoreactivity in whole mount preparation of a cerebral artery. Double immunostaining of the VPAC1 receptor (green) and VIP (red) in panel **(A)** and triple immunostaining of the same artery in panel **(B)** with CGRP (blue). Note that the two neuropeptides have different distribution patterns. Higher magnification illustrates VPAC1 immunostaining on the surface of smooth muscle cells innervated by VIP-containing nerve fibers **(C,D)**. VIP immunoreactive nerve fibers with close contact to the VPAC1 receptor on the plasmalemma of cross-sectioned smooth muscle cells in panel **(C)**. Cross section of a basal artery of wild type mouse close to a. cerebri ant. shows no expression of the PAC1 receptor (green) **(E,G)** or the VPAC2 receptor (green) **(F,H)**. Note that in PAC1 **(G)**—and in VPAC2 **(H)** deficient mice no specific staining can be demonstrated as also found in both wild type mice [PAC1 in panel **(E)** and VPAC2 in panel **(F)**]. Panels **(A,B)** represent a 3D reconstruction of a Z-stack [**(A)**: Z = 0.5 μm, **(B)**: Z = 0.3 μm] of 152 digital sections. Scale bars **(A,B)** = 20 μm, **(C)** = 10 μm, **(D)** = 20 μm, **(E–H)** = 30 μm.

Whole-mount preparation of the basal cerebral arteries demonstrated a dense plexus of perivascular VIP-containing nerve fibers in the wall of the cerebral arteries with the highest density in the anterior part of the circle of Willis (Figure 1). The VIP-fibers were abundant and mainly circularly oriented. Double and triple immunostaining visualized that PACAP and CGRP were in different nerve fibers than VIP (Figures 1C,E,F and Supplementary Animation 1). PACAP nerve fibers were found primarily along the long axis of the blood vessels with a few horizontal branches. PACAP and CGRP appeared to be colocalized in nerve fibers (Figure 1D). Using well-characterized antibodies raised against the PAC1 (Figures 2E,G; Hannibal et al., 2017b), VPAC1 (Figures 2A–D; Fahrenkrug et al., 2000), and VPAC2 (Figures 2F,H; Hannibal et al., 2017b) receptors, we discovered that only the VPAC1 receptor could be found in the basal arteries (Figures 2A–D). VPAC1 immunostaining showed a distinct pattern with immunoreactivity in the membrane of circularly oriented smooth muscle cells (Figures 2C,D). VIP fibers with characteristic synaptic boutons in passing were found near VPAC1 immunoreactivity. 3D analysis showed discrete colocalization of VIP and VPAC1 suggesting synaptic appositions (Figures 2A,B and Supplementary Animation 2). No immunostaining was observed in the endothelial cell layer of the blood vessels.

### Pituitary adenylate cyclase-activating polypeptide, vasoactive intestinal peptide, and calcitonin gene-related peptide in mouse trigeminal ganglion

**FIGURE 3:**
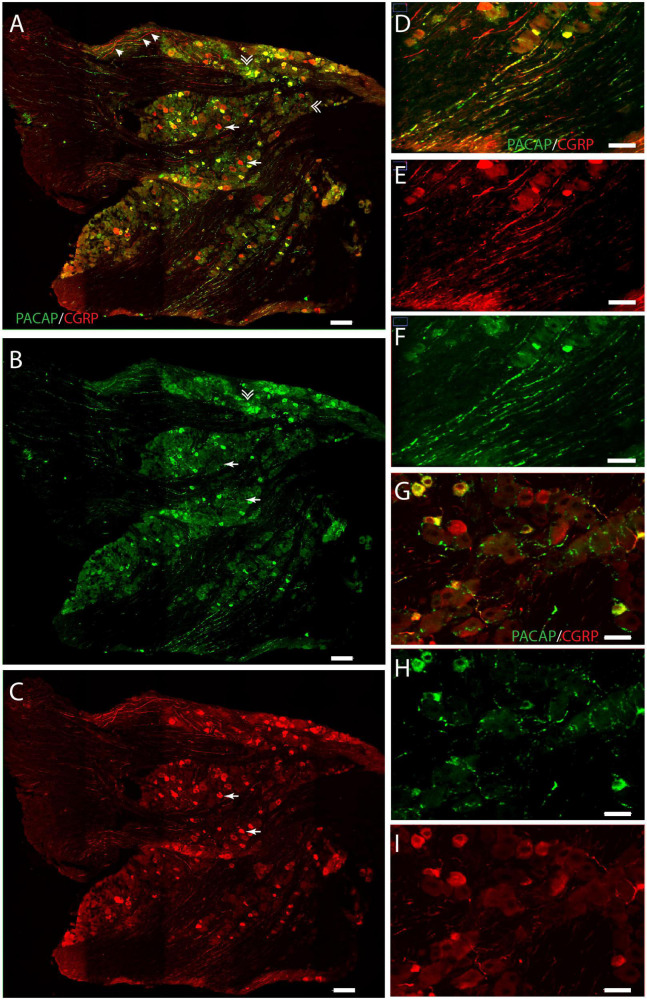
Distribution of PACAP (green) and CGRP (red) immunoreactivity in the trigeminal ganglion. Panels **(A–C)** provide an overview with double immunostaining **(A)**, PACAP **(B)**, and CGRP **(C)**. Arrows in panel **(A)** indicate cells expressing solely CGRP, double arrows indicate cells expressing only PACAP, and arrow tips show immunoreactive nerve fibers. Higher magnification in panel **(D–F)** visualizes nerve fibers with double immunostaining **(D)**, CGRP **(E)**, and PACAP **(F)**. PACAP-positive delicate nerve fibers between neurons in close apposition, suggesting innervation **(G–I)**. Scale bars **(A–C)** = 100 μm, **(D–F)** = 50 μm, **(G–I)** = 30 μm.

**FIGURE 4:**
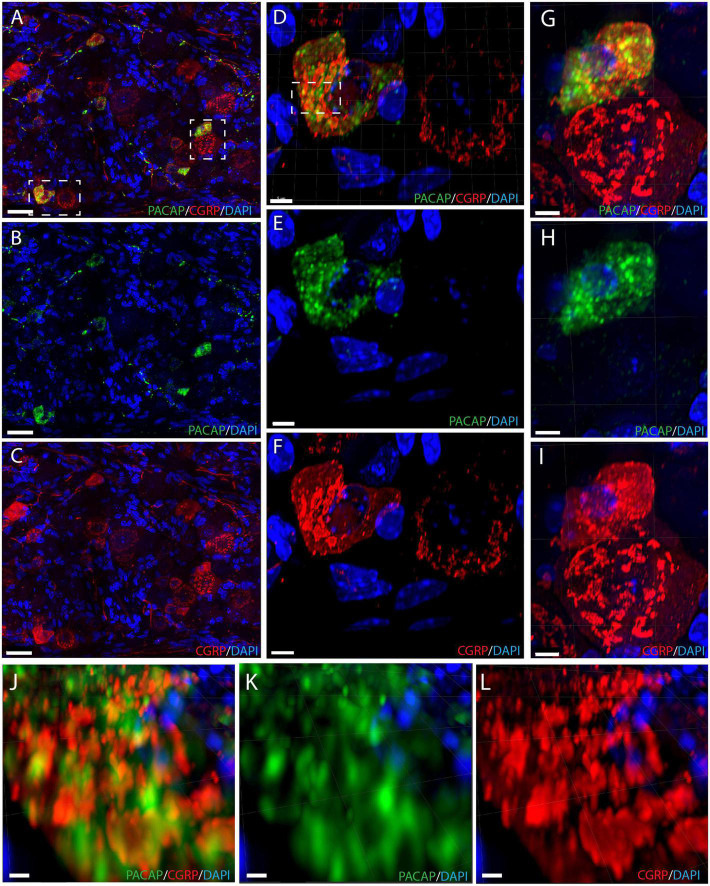
Immunostaining showing neuronal somas with intracellular components in the trigeminal ganglion **(A–C)** with PACAP (green) and CGRP (red). Double immunostaining in panel **(A)**, PACAP **(B)**, and CGRP **(C)**. The left dashed box in panel **(A)** is magnified in panels **(D–F)** and shows a cell with colocalized neuropeptides and a CGRP-positive cell. Double immunostaining is seen in panel **(D)**, PACAP **(E)**, and CGRP **(F)**. The right dashed box in panel **(A)** is magnified in panels **(G–I)** and illustrates another colocalized cell and a CGRP-positive cell with double immunostaining **(G)**, PACAP **(H)**, and CGRP **(I)**. The dashed box in panel **(D)** is magnified in panels **(J–L)** illustrating distinct secretory granules containing either PACAP or CGRP in a cell with colocalization. Double immunostaining in panel **(J)**, PACAP **(K)**, and CGRP **(L)**. Scale bars **(A–C)** = 30 μm, **(D–I)** = 5 μm, **(J–L)** = 2 μm.

We found that the neuronal somas in the trigeminal ganglion varied in size and were typically located in a cluster next to the three major nerve branches (ophthalmic, maxillary, and mandibular) or in some cases in smaller bundles of neurons in between nerve fibers (Figure 3). Immunostaining demonstrated PACAP in small to midsized neurons (12–15 μm in diameter) distributed in all parts of the ganglia (Figures 3A,B). Within the single neuron, PACAP was localized in what seemed to be secretory granules in the cell cytoplasm (Figures 4E,H). PACAP-positive neuronal fibers were seen as fibers passing through the major branches of the trigeminal nerve (Figures 3D,F) and as more delicate nerve fibers between the ganglionic neurons indicating innervation (Figures 3G,H). Double immunostaining revealed that nearly all PACAP immunoreactive nerve cells contained CGRP, representing a subpopulation of CGRP-expressing neurons. Corresponding to the degree of colocalization in neuronal somas, numerous nerve fibers co-stored PACAP and CGRP. An average of 1–3 neuronal somas and nerve fibers per slide expressed solely PACAP. Many nerve fibers contained only CGRP in agreement with a large number of CGRP-expressing neurons (Table 3) found without PACAP (Figures 3, 4). These neurons were larger than cells with colocalization, approximately 28–42 μm in diameter (Figures 4A,D,G). High-resolution microscopy and 3D analysis of neurons co-storing CGRP and PACAP revealed that the neuropeptides appeared in different subcellular vesicles in the cell cytoplasm (Figures 4J–L). Interestingly, neurons only expressing CGRP seemed to store CGRP-immunoreactivity in larger subcellular granules compared to the granules found in neurons co-storing CGRP and PACAP (Figures 4D,G). Cell counting in the mouse trigeminal ganglion showed that cells containing only CGRP represented 66% of all immunoreactive cells counted, whereas 33% co-stored PACAP and CGRP. Only 1.4% of the counted neurons expressed solely PACAP (Table 3). A few VIP nerve fibers were found in the major branches of the trigeminal ganglion, while no cell bodies expressed VIP. Immunostaining of PACAP and VIP receptors in the trigeminal ganglion using antibodies for the PAC1, VPAC1, and VPAC2 receptors suggested that there was no detectable specific receptor staining in in cell bodies or nerve fibers (Supplementary Figures 2–4).

### Pituitary adenylate cyclase-activating polypeptide and the PAC1 receptor in SP5

**FIGURE 5:**
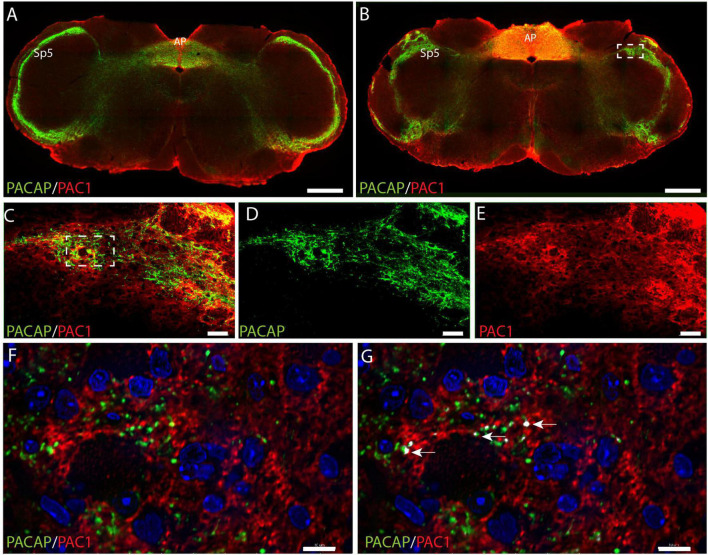
Distribution of PACAP (green) and PAC1 (red) at two levels of the brain stem at the level of the spinal trigeminal nucleus (SP5) and area postrema (AP). An overview of the brain stem in two different levels is presented in panels **(A,B)**. The dashed box in panel **(B)** is magnified in panels **(C–E)** with double immunostaining in panel **(C)**, PACAP **(D)**, and PAC1 **(E)**. The dashed box in panel **(C)** is further magnified in panels **(F,G)** showing PAC1 immunoreactivity near PACAP nerve terminals. The arrows in panel **(G)** represent close appositions (colocalization) visualized with IMARIS colocalization module, most likely representing synaptic appositions. Scale bars **(A,B)** = 500 μm, **(C–E)** = 50 μm, **(F,G)** = 10 μm.

Nerve cell bodies in the trigeminal ganglion are considered sensory nerve cells with axons terminating in the spinal trigeminal nucleus (SP5) located in the lateral medulla of the brain stem. Dense innervation of SP5 with PACAP immunoreactive nerve fibers was found in the entire rostro-caudal axis of the nucleus (Figures 5A,B and Supplementary Animation 3). In the same area of the SP5, PAC1 immunoreactivity was found in what seemed to be the cell membrane of nerve cell bodies and their processes (Figures 5C–E and Supplementary Animation 3). High-resolution images in 3D were deconvoluted and analyzed with IMARIS using the colocalization module (Hannibal et al., 2017a). There were several points of colocalization, most likely representing synaptic appositions (Figures 5F,G).

## Discussion

To our knowledge, this is the first study to show the distribution of nerve fibers expressing PACAP and VIP in the basal arteries of the mouse and the localization of the VPAC1 receptor in the smooth muscle cells of these arteries. The distribution of both peptides and the VPAC1 receptor is similar to what has previously been described in the rat (Fahrenkrug et al., 2000). Furthermore, PACAP nerve fibers were found to co-express CGRP. PACAP was primarily stored in a subpopulation of CGRP neurons located in the trigeminal ganglion, supporting a sensory role of these two peptides in the vasoregulation of the basal arteries. This notion is supported by the expression of the PAC1 receptor in neurons of the SP5 nucleus in the brain stem, densely innervated by PACAP nerve terminals, and the observation that neither the PAC1 nor the VPAC2 receptor could be demonstrated on basal arteries.

The distribution of PACAP and VIP fibers in the basal arteries has been studied previously in other species but with different results. A study in cat cerebral arteries discovered that the majority of PACAP fibers in the circle of Willis contained VIP and a subpopulation of VIP fibers lacked PACAP (Uddman et al., 1993). In contrast with these findings, a study in rat arteries observed no colocalization of the two neuropeptides in nerve fibers using the same antibodies as in the present study (Fahrenkrug et al., 2000). VIP fibers formed a circularly oriented network, whereas PACAP was found in fewer fibers following the longitudinal axis of the vessels (Fahrenkrug et al., 2000). Our findings correspond with the previous study in the rat (Fahrenkrug et al., 2000). Furthermore, in the present study, we used triple immunostaining to visualize CGRP in relation to the distribution of PACAP and VIP and discovered that CGRP was present in PACAP nerve fibers on the cerebral blood vessels. This corresponds with our findings in the trigeminal ganglion where PACAP was found in a subpopulation of CGRP-expressing neurons, indicating that the trigeminal ganglion is the origin of PACAP fibers on the blood vessels. Previous studies have demonstrated PACAP nerve cells in sympathetic ganglia, parasympathetic ganglia (Sundler et al., 2006), and in the sensory trigeminal ganglion where it colocalizes with CGRP (Eftekhari et al., 2015), and retrograde tracing from rat cerebral blood vessels combined with immunohistochemistry found PACAP predominantly in the trigeminal ganglion (Martin et al., 2004). We identified relatively few nerve fibers and cell bodies expressing exclusively PACAP in the trigeminal ganglion. These findings could indicate that the expression of CGRP is too low to be detected in these PACAP cells or that the few PACAP neurons in the trigeminal ganglion belong to a distinct population projecting to other structures. Furthermore, we found a few VIP-containing nerve fibers in the trigeminal ganglion but the lack of VIP receptors in the ganglion does not support a role in this structure. VIP nerve cell bodies innervating cerebral blood vessels are known to originate in parasympathetic ganglia (sphenopalatine and otic) (Suzuki et al., 1988; Hara et al., 1989). Therefore, the VIP fibers from these ganglia could be passing through the trigeminal ganglion, having a function in other tissues.

A few studies have performed cell counting in the trigeminal ganglion. A study in the rhesus monkey and in the rat found that CGRP-only cells represented 46% of all immunoreactive cells, 10% PACAP-only, and 44% colocalizing (Eftekhari et al., 2015). Another study in rats identified 68% CGRP-only cells, 23% PACAP-only cells, and 9% of the immunoreactive cells co-storing CGRP and PACAP (Edvinsson et al., 2020). We found 66% CGRP-only, 1.4% PACAP-only, and 33% colocalizing both peptides. Antibody specificities or species differences between mice, rats, and monkeys could be a potential explanation for the discrepancy.

High-resolution, deconvolution, and 3D analysis in our study revealed that the subcellular distribution was different for CGRP and PACAP in the trigeminal ganglion, indicating that PACAP and CGRP could be released independently. This notion is supported by several studies in mice. One study found that PACAP dilated carotid arteries and induced increased hypersensitivity independent of CGRP (Ernstsen et al., 2022). Another study investigated light aversion as a behavioral surrogate for migraine photophobia and observed that both PACAP and CGRP induced light aversion in mice (Kuburas et al., 2021). Furthermore, a CGRP monoclonal antibody could block CGRP-induced light aversion but not PACAP-induced, and equally a PACAP antibody could not reduce CGRP-induced light aversion.

Pituitary adenylate cyclase-activating polypeptide and CGRP are co-stored in many tissues and are believed to be sensory neuropeptides. PACAP has been demonstrated in the rat in a subpopulation of CGRP/substance P (SP) immunoreactive fibers in the dorsal horn of the spinal cord, dorsal root (spinal) ganglia, trigeminal ganglia (Moller et al., 1993), and in other tissues including the gastrointestinal tract and urogenital tract (Moller et al., 1993; Fahrenkrug and Hannibal, 1996, 1998; Hannibal et al., 1997b). CGRP is mainly localized in sensory, unmyelinated C fibers that are capsaicin-sensitive due to a high degree of capsaicin receptor expression (Ilie et al., 2019). Capsaicin has gained a superior position in the investigation of primary afferent neurons due to its selective neurotoxic effect on C fibers (Ilie et al., 2019). Cell bodies colocalizing CGRP and substance P have been found in the sensory ganglia of the rat (trigeminal and dorsal root) and were markedly depleted by capsaicin treatment (Skofitsch and Jacobowitz, 1985). Another study showed that capsaicin treatment reduced the density of immunoreactive fibers co-storing PACAP and CGRP/SP in the dorsal horn of the spinal cord, dorsal root ganglia, and trigeminal ganglia (Moller et al., 1993). In the gastrointestinal and urogenital tracts, the vast majority of PACAP fibers also contained CGRP and these nerve fibers were sensitive to capsaicin (Fahrenkrug and Hannibal, 1996, 1998; Hannibal et al., 1997b). These findings indicate that nerve fibers co-storing CGRP and PACAP with neuronal somas located in sensory ganglia are capsaicin-sensitive. It has not been determined whether the nerve fibers on the basal blood vessels are capsaicin-sensitive, but the localization of PACAP and CGRP in the same nerve fibers of these vessels with a probable origin in the trigeminal ganglion supports this notion.

Sensory information is generally thought to travel from the peripheral terminals of primary afferent neurons to the CNS. However, there is evidence that release of neuropeptides can be generated by peripheral or central stimulation (Lobanov and Peng, 2011; Gafurov et al., 2021). Release of CGRP and SP from C fibers has been shown to contribute to neurogenic inflammation (Maggi, 1995; Sousa-Valente and Brain, 2018). PACAP expression is upregulated in neurons of the dorsal root ganglia within 24 h of axonal injury (Jongsma et al., 2000), transported to the regenerating nerve, and released (Reimer et al., 1999). The release of PACAP from peripheral nerve endings in the basal blood vessels would require a receptor to mediate the effect of the neuropeptide. *In situ* hybridization in rat cerebral arteries has found mRNA of all three receptors in the smooth muscle cells of the vessels with a predominance of the VPAC1 receptor over VPAC2 and PAC1 (Baun et al., 2011), and the same pattern was seen in a different study in human tissue using qPCR (Chan et al., 2011). However, using VPAC2 antagonist and PAC1 receptor agonist did not support a role for either of these receptors in the relaxation induced by VIP or PACAP (Baun et al., 2011). We found no staining for the PAC1 and VPAC2 receptors in the cerebral blood vessels of the mouse using specific antibodies (Hannibal et al., 2017b). Immunostaining of the VPAC1 receptor was demonstrated on the surface of vascular smooth muscle cells in proximity to VIP fibers, as previously described in the rat (Fahrenkrug et al., 2000). Anatomically, this could indicate that the effect of both VIP and PACAP occurs is via the VPAC1 receptor in the basal arteries, but the timeline for induction of migraine after VIP and PACAP infusion is quite different, suggesting separate receptors and downstream pathways. A 20-minute infusion of PACAP induced delayed migraine attacks in migraine patients 4-5 hours after the termination of the infusion (Schytz et al., 2009) while two hours of continuous VIP infusion was necessary to generate migraine attacks (Pellesi et al., 2021). If the effect of PACAP is not via the VPAC1 receptor, another explanation could be that PACAP stimulates the PAC1 and VPAC2 receptors expressed on immune cells in the meninges. Alternatively, PACAP may activate an orphan receptor as was demonstrated recently in meningeal mast cells where PACAP was shown to stimulate mast cell degranulation via a mechanism involving orphan receptor MrgB_3_ independent of the PAC1 receptor (Pedersen et al., 2019). The PAC1 receptor gene is expressed in rat peritoneal macrophages and microglia (Pozo et al., 1997) and the VPAC2 receptor in macrophages and lymphocytes in mice upon stimulation (Miller, 2006; Abad and Tan, 2018). The trigeminal innervation of cerebral blood vessels and meninges, forming the trigeminovascular system, is believed to be the origin of migraine pain (Messlinger, 2009; Gafurov et al., 2021). Therefore, the release of PACAP from trigeminal nerve fibers might initiate neurogenic inflammation associated with migraine pathology. More insight into the release of PACAP and the type of nerve fibers in the basal blood vessels is needed.

mRNA of the PAC1, VPAC1, and VPAC2 receptors has been found in the trigeminal ganglion of rats and humans (Chaudhary and Baumann, 2002; Knutsson and Edvinsson, 2002; Frederiksen et al., 2018), but we found no staining of the three receptors. We have used a detection system based on tyramide (ENVISION and/or biotinylated tyramide), which had two major advantages. First, the signal is strongly amplified and the signal to noise is improved (Berghorn et al., 1994) and second, saving primary antibodies, and in case of polyclonal antibodies enhancement of selective dominant clones. Despite using powerful amplification systems, the level of receptor protein could be too low to be detected by the methods used (Supplmentary Figures 2–4). However, investigation on primary cultures of rat and mouse trigeminal cells and calcium imaging techniques demonstrates that VIP and PACAP (both 1–27 and 1–38 isoforms) and the PACAP agonist maxadilan cause an increase in intracellular calcium concentration. Similar response was found following the administration of the PACAP antagonists PACAP6-38 and M65 and the VPAC2 receptor antagonist BAY 55-9837, while the VPAC1 receptor antagonist Ala(11,22,28) VIP had no effect (Sághy et al., 2015). The studies shows that the antagonists may not always act entirely as antagonist as found in other systems (Németh et al., 2006; Reglodi et al., 2008), and open for the existents of unknown splice variants of the receptors using different signaling transduction pathways or alternatively, the existence of unknown or orphan receptors in the trigeminal ganglion neurons. Distinct PAC1 immunostaining was found in neurons of the SP5 nucleus in the brain stem, densely innervated by PACAP fibers, supporting that PACAP-expressing neurons in the trigeminal ganglion project to the brain stem and verify the affinity and specificity of the PAC1 antibody used in our study (see also Supplementary material and Supplementary Figure 1). Nerve cells in the dorsal root ganglia in rats did not contain PAC1 mRNA apart from a few neurons (less than 1%) (Jongsma et al., 2000). The dorsal root ganglia and trigeminal ganglia consist of pseudo unipolar sensory neurons and serve similar roles of encoding somatosensory modalities (Messlinger et al., 2020). The expression of PACAP in neurons of the dorsal root ganglia resembles the trigeminal ganglion (Moller et al., 1993), and therefore it seems plausible that the distribution of the PAC1 receptor is alike.

## Conclusion

The present study demonstrates that the VPAC1 receptor is present in the basal arteries on the surface of circularly oriented smooth muscle cells, densely innervated by VIP fibers. This indicates that the effect of VIP occurs via the VPAC1 receptor, while no staining was seen for the PAC1 or VPAC2 receptors. PACAP was found in a subpopulation of CGRP-expressing neurons in the trigeminal ganglion and colocalized with CGRP in nerve fibers on the basal arteries. This localization supports that PACAP has a primary sensory function in basal arteries, although an effect via release of the transmitter could be involved in vasodilatation and neurogenic inflammation relevant for migraine pathology. The effect of PACAP might occur via the VPAC1 receptor on basal arteries or possibly via immune cells in the meninges expressing the PAC1 and VPAC2 receptors or an orphan receptor.

## Data availability statement

The original contributions presented in this study are included in the article/Supplementary material, further inquiries can be directed to the corresponding author.

## Ethics statement

This animal study was reviewed and approved by Dyreforsoegstilsynet, Ministry of Justice, Denmark. Written informed consent was obtained from the owners for the participation of their animals in this study.

## Author contributions

AL and JH: conceptualization, formal analysis, wrote the final version, and approve the submitted version. JH: methods, imaging, figures, and writing. AL: draft the manuscript and contributed to the analysis.
