# Supplementary material for: Localization of the neuropeptides pituitary adenylate cyclase-activating polypeptide, vasoactive intestinal peptide, and their receptors in the basal brain blood vessels and trigeminal ganglion of the mouse CNS; an immunohistochemical study
Source: Front Neuroanat. 2022 Oct 26;16:991403. doi: 10.3389/fnana.2022.991403 (PMC9643199; doi:10.3389/fnana.2022.991403)
Supplement: Supplementary file 1 [file Data_Sheet_1.pdf]

Supplementary material

Supplementary Animation 1: This video illustrates a rotating 3D-reconstruction of a blood vessel from the circle of Willis with DAPI-staining (grey), CGRP (blue), PACAP (green), and VIP (red). Immunostaining for CGRP, PACAP, and VIP is added once at a time to visualize the distinct distribution of the neuropeptides. Note that CGRP and PACAP are localized in the same nerve fibers but in different transmitter vesicles. Scale bar: 40 μm

Supplementary Animation 2: This video illustrates a rotating 3D-reconstruction of a blood vessel from the circle of Willis with DAPI-staining (blue), VPAC1 (green), VIP (red), and CGRP (grey). Immunostaining for VPAC1, VIP, and CGRP is added once at a time to visualize the distinct distribution of the neuropeptides and receptors. Note that VIP-positive nerve fibers innervate the VPAC1 receptor. Scale bar: 50 μm

Supplementary Animation 3: This video illustrates a rotating 3D-reconstruction of one neuron located in the SP5 stained by DAPI (blue) showing the cell nucleus and in red PAC1 receptor immunoreactivity (red) most likely located in the cell membrane and PACAP nerve terminals (green) in close apposition to the PAC1 receptor (white). Scale bar 5 μm.

Supplementary Material and methods

The brain, CW and TG from two mice of each genotype, lacking either the VPAC2 or the PAC1 receptor (Hannibal, et al. 2017b), was used as negative controls for validation of the PAC1 – and the VPAC2 receptor antibodies. Mice were perfusion-fixed, dehydrated in 30 % sucrose, frozen, and the brains were cut into 40 μm sections and stored in cryoprotectant until

immunohistochemical processing, while CW were removed as described in the main text and the TG cut in 12 µm thick sections on slides. All tissue sections and CW were treated in heat-induced antigen retrieval at 80°C with EnVision Flex GV805 in distilled water pH 6 for 1 hour and 30 minutes. The tissues were then washed (PBS/ 0.25% Triton X-100), blocked for endogenous peroxidase activity (1% H_2O_2 in 1xPBS), and subsequently blocked with 5% normal donkey serum (Jackson Immunoresearch Laboratories) to avoid non-specific staining. Hereafter, the tissues were incubated with primary rabbit polyclonal antibodies directed against the PAC1 – and VPAC2 receptor (Table 1). For the routine validation of the primary antibodies used in the study, omission of the antibodies was performed using the respective detection systems described in the main text (example of immunostaining for the VPAC1 receptor is shown in supplementary fig. 4).

In the case of CGRP, both antibodies are commercially available, raised in different species, used by many researchers (published previously) and by us for years. The localization of the protein has been confirmed by the localization used in in situ hybridization histochemistry for the localization of the mRNA and such signals can be compared with that found in Allen's Brain Atlas from the mouse brain (https://mouse.brain-map.org/).

Results

Immunostaining using antibodies directed against the PAC1,- the VPAC1, - and the VPAC2 receptor demonstrated that in the tissue from PAC1 deficient mice no specific staining can be found while specific staining is exemplified in the mouse SCN (Suppl. Fig 1 A-B) and similar using the VPAC2 receptor antibody on mouse SCN tissue from wild type and VPAC2 deficient mice (Suppl. Fig. 1C-D). Staining of the mouse trigeminal ganglion using the PAC1 and the VPAC2 receptor antibody of wild type and receptor deficient mice reveal that none of the receptors can be detected in the TG (Suppl. Fig. 2-3). However, a slight background staining

using the PAC1 receptor antibody can be found in what seems to be nerve fibres in the TG in both wild type and PAC1 receptor deficient mice (Suppl. Fig. 2 E-F). Omission of the primary antibody demonstrated in the TG using the VPAC1 antibody that no specific staining is related to the detection system (Suppl. Fig. 4).

Figures and figures legends

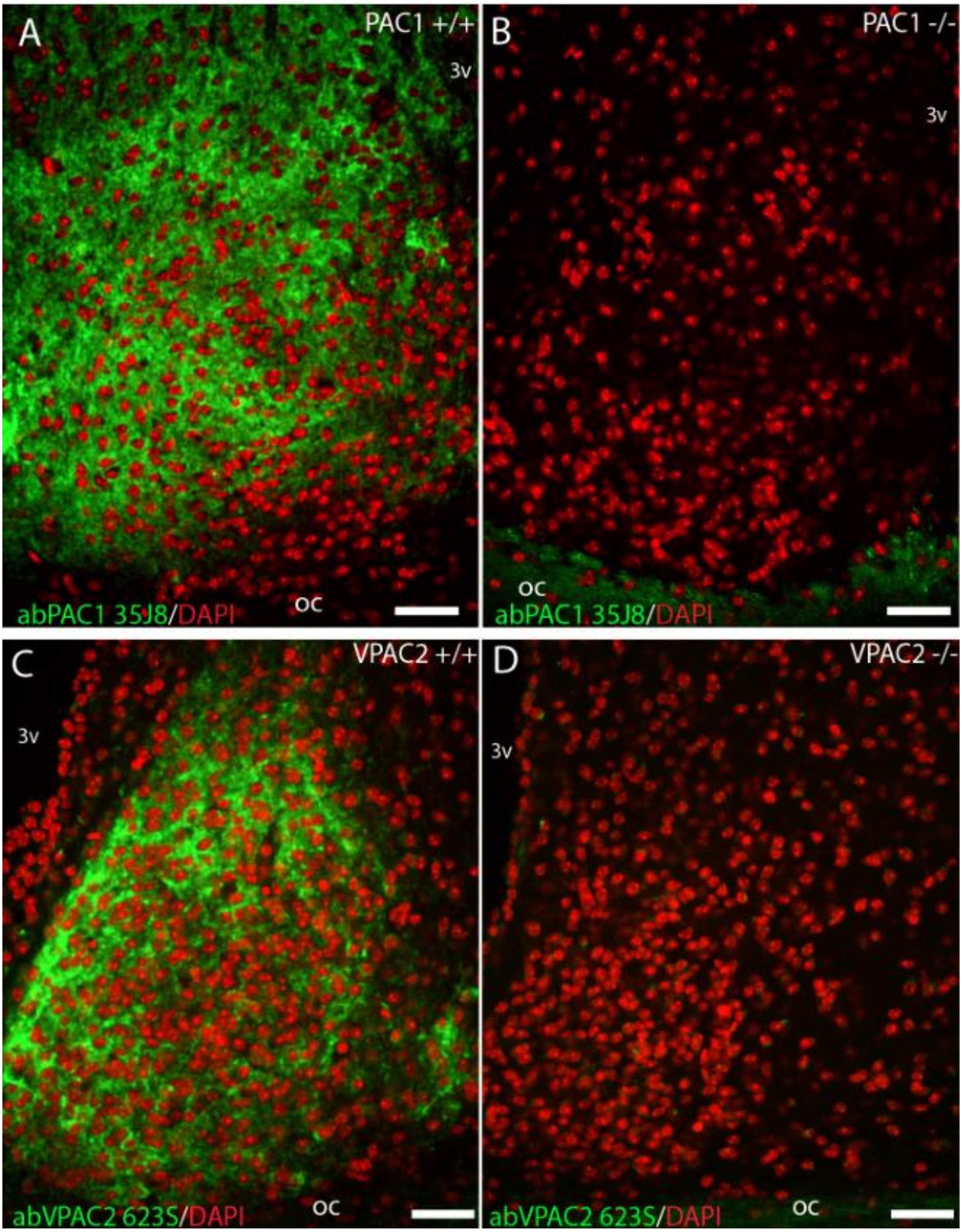

Supplementary Figure 1.

Immunostaining of the PAC1 (Panel A-B) and VPAC2 receptors (Panel C-D) in the mouse SCN were used to demonstrate the specificity of the receptor staining in a well described area of the brain (Hannibal et al. 2017b). Specific staining of the PAC1 receptor was found in the retinorecipent zone of the SCN (Suppl. Fig 1A), while VPAC2 receptor immunoreactivity was found mainly in the core and shell of the SCN (Suppl. Fig 1C) in wild type mice. No specific staining of either the PAC1 receptor (B) or the VPAC2 receptor (D) was found in the knockout mice, respectively (Suppl. Fig. 1B and D). Scale bars; 50 μm, oc; optic chiasm, 3v; third ventricle.

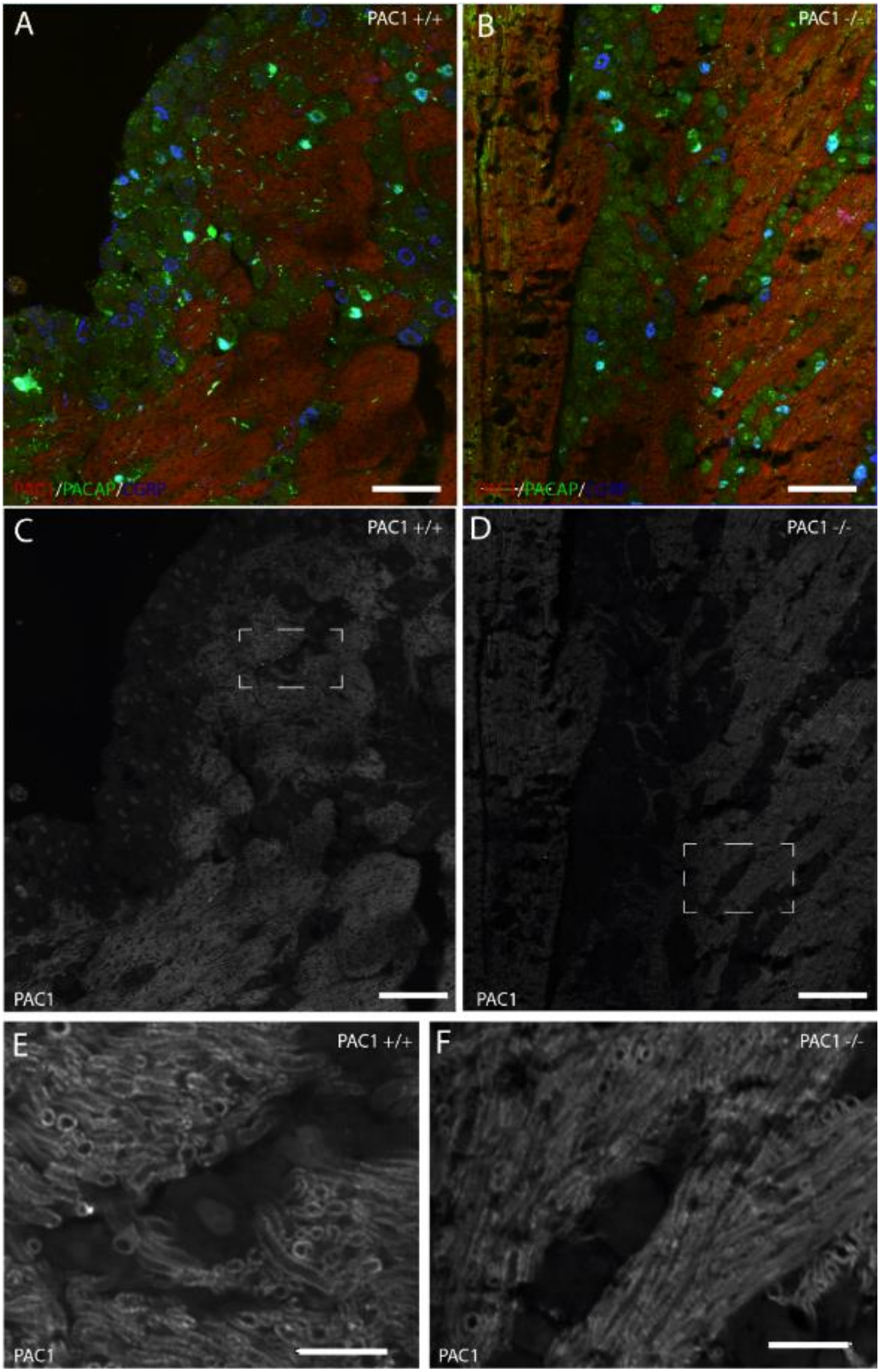

A
PAC1 +/+
PAC1/PACAP/CGRP
B
PAC1 -/-
PAC1/PACAP/CGRP
C
PAC1 +/+
PAC1
D
PAC1 -/-
PAC1
E
PAC1 +/+
PAC1
F
PAC1 -/-
PAC1

Supplementary Figure 2

Immunostaining of the PAC1 receptor in the trigeminal ganglion (TG) in PAC1 wild type mice (A and C) and PAC1 deficient mice (B and D) together with PACAP (green) and CGRP (blue)(A-B), shown PACAP and CGRP in neuron and in nerve fibres in the TG while no specific staining was found for the PAC1 receptor (red in A-B, white in C-F) in neither the wild type nor in the KO mice. Insert in C and D are shown in high magnification in E and F showing a slightly higher background in what seems to be nerve fibres in the TG in both wild type – and PAC1 deficient mice. Scale bars; A-D; 100 μm, E-F; 25 μm

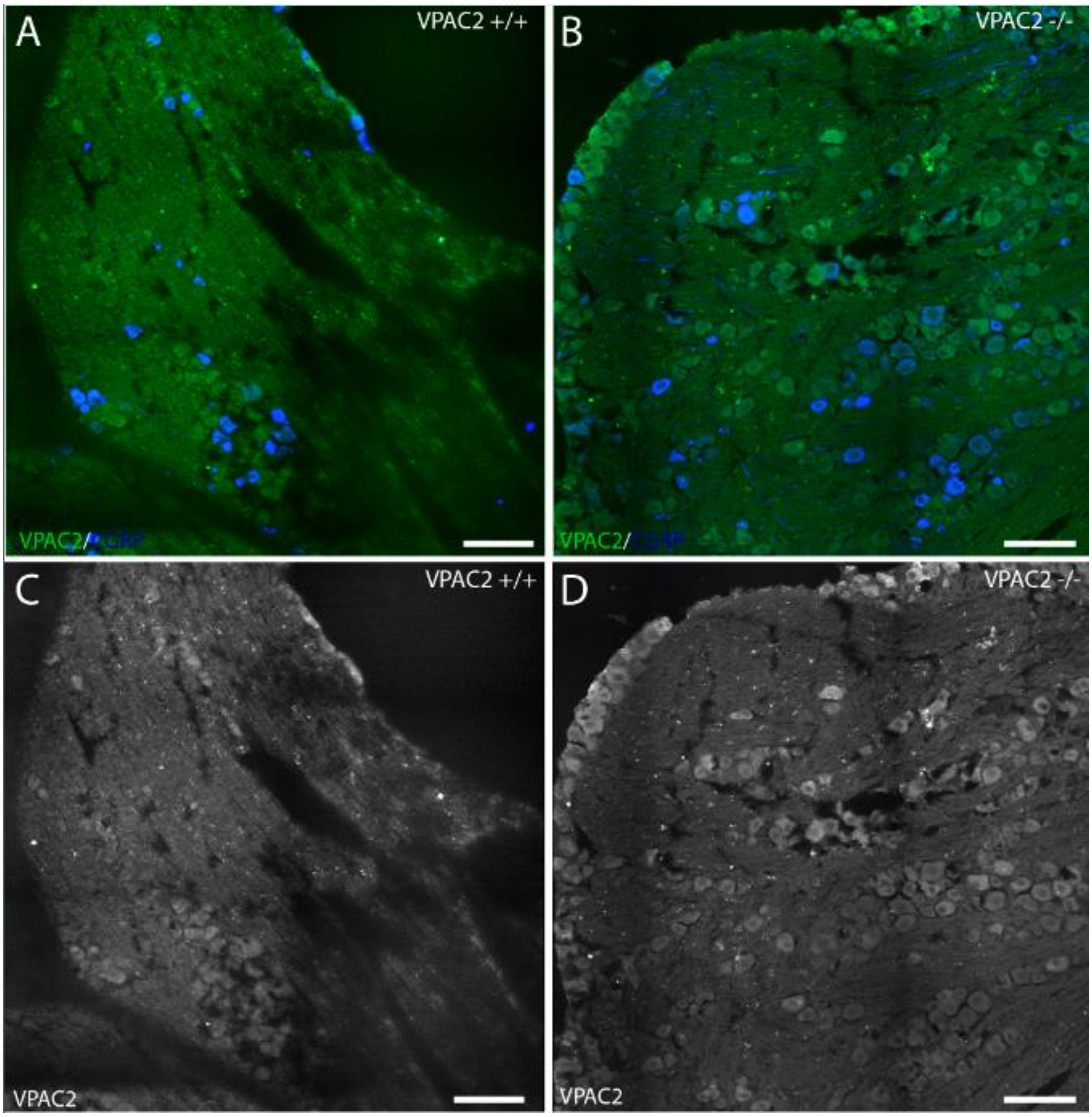

Supplementary Figure 3

Immunostaining of the VPAC2 receptor in the trigeminal ganglion (TG)(green) in VPAC2 wild type - (A and C) and VPAC2 deficient mice (B and D) together with CGRP (blue)(A-B), shows CGRP in the neuron and in the nerve fibres in the TG while no specific staining was found for the VPAC2 receptor (green in A and B, white in C-D) of neither the wild type (A and C) nr the KO mice (B and D). Scale bars; 100 µm.

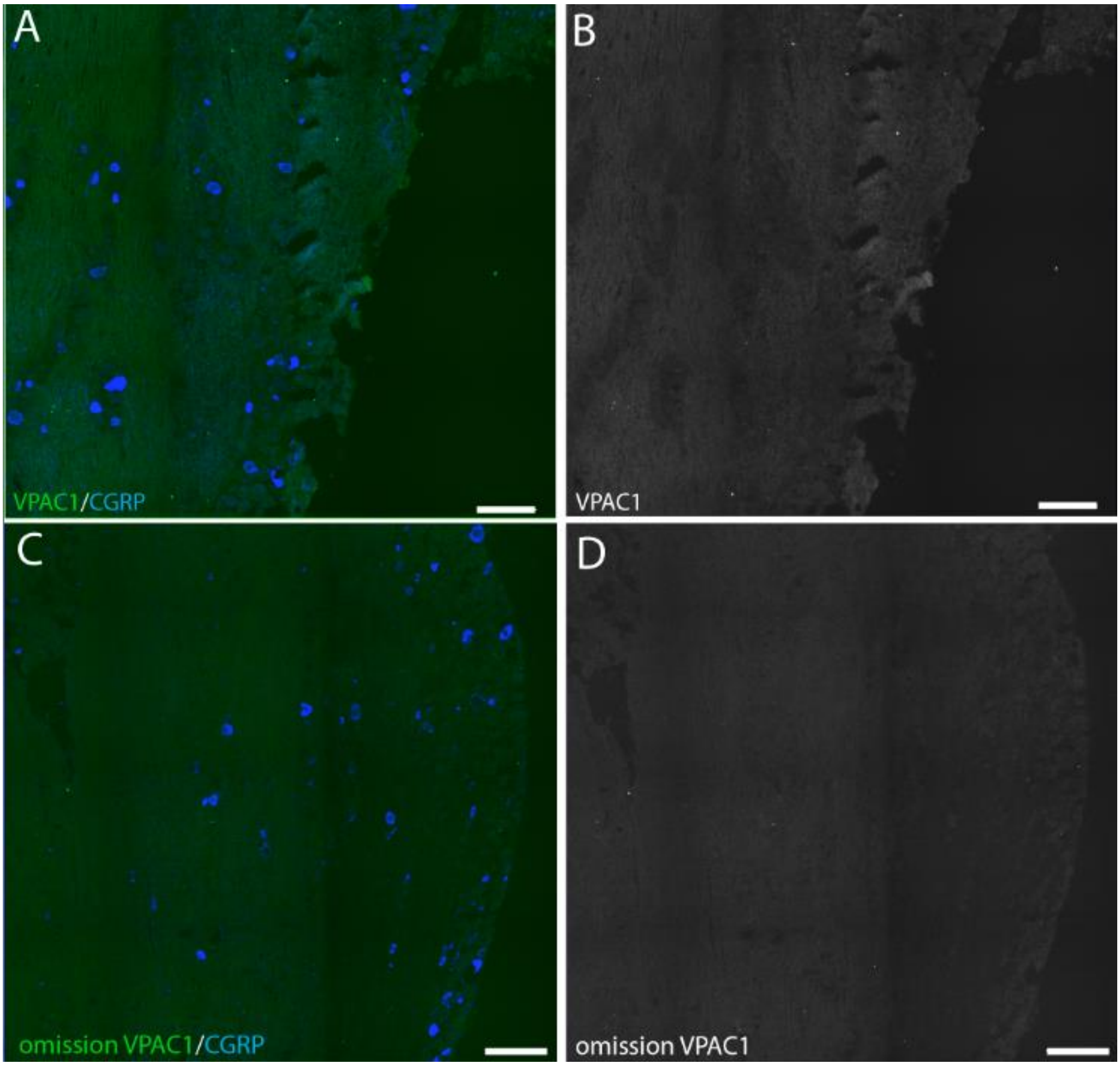

Supplementary Figure 4

Immunostaining of the VPAC1 receptor (green) and CGRP (blue) in the trigeminal ganglion (TG) in wild type mice. CGRP was found in neuron and in nerve fibers in the TG (blue in A and C) while no specific staining was found for the VPAC1 receptor in the TG (A and B). Omission of the primary antibody (VPAC1) confirmed no specific staining of VPAC1 in the TG and no unspecific staining from the detection system (ENVISION) (C and D). Scale bars; 100 μm.
